# Supplementary material for: Time-series transcriptome analysis identified differentially expressed genes in broiler chicken infected with mixed Eimeria species
Source: Front Genet. 2022 Aug 8;13:886781. doi: 10.3389/fgene.2022.886781 (PMC9393255; doi:10.3389/fgene.2022.886781)
Supplement: Supplementary file 2 [file DataSheet1.ZIP › 7dpi_KEGG.Gsea.1625115961950/KEGG_NITROGEN_METABOLISM.html]

Details for gene set KEGG\_NITROGEN\_METABOLISM[GSEA]

|  || Dataset | TMM\_7dpi\_gct\_format\_7dpi\_gct\_format.Class\_4dpi.cls #PC\_versus\_NC.Class\_4dpi.cls #PC\_versus\_NC\_repos |
| Phenotype | Class\_4dpi.cls#PC\_versus\_NC\_repos |
| Upregulated in class | 1 |
| GeneSet | KEGG\_NITROGEN\_METABOLISM |
| Enrichment Score (ES) | 0.399368 |
| Normalized Enrichment Score (NES) | 1.3288355 |
| Nominal p-value | 0.11330935 |
| FDR q-value | 0.29840958 |
| FWER p-Value | 0.9386 |
Table: GSEA Results Summary

  

Fig 1: Enrichment plot: KEGG\_NITROGEN\_METABOLISM      
 Profile of the Running ES Score & Positions of GeneSet Members on the Rank Ordered List

  

| SYMBOL | TITLE | RANK IN GENE LIST | RANK METRIC SCORE | RUNNING ES | CORE ENRICHMENT || 1 | CA2 | na | 14 | 1.747 | 0.1620 | Yes |
| 2 | CA8 | na | 59 | 1.398 | 0.2888 | Yes |
| 3 | CA9 | na | 166 | 1.102 | 0.3828 | Yes |
| 4 | GLS2 | na | 687 | 0.641 | 0.3994 | Yes |
| 5 | AMT | na | 1224 | 0.448 | 0.3965 | No |
| 6 | CTH | na | 1955 | 0.278 | 0.3616 | No |
| 7 | HAL | na | 2259 | 0.228 | 0.3576 | No |
| 8 | CA13 | na | 3131 | 0.110 | 0.2953 | No |
| 9 | GLUL | na | 4227 | -0.021 | 0.2060 | No |
| 10 | GLUD2 | na | 4781 | -0.078 | 0.1672 | No |
| 11 | CPS1 | na | 5072 | -0.110 | 0.1532 | No |
| 12 | ASNS | na | 5766 | -0.177 | 0.1120 | No |
| 13 | CA5A | na | 5804 | -0.180 | 0.1256 | No |
| 14 | CA6 | na | 6897 | -0.297 | 0.0623 | No |
| 15 | CA7 | na | 11960 | -1.704 | -0.2006 | No |
| 16 | CA4 | na | 12001 | -2.192 | 0.0008 | No |
Table: GSEA details [plain text format]

  

Fig 2: KEGG\_NITROGEN\_METABOLISM      
 Blue-Pink O' Gram in the Space of the Analyzed GeneSet

  

Fig 3: KEGG\_NITROGEN\_METABOLISM: Random ES distribution      
 Gene set null distribution of ES for **KEGG\_NITROGEN\_METABOLISM**
